# Supplementary material for: Intergenerational Transmission of Gut Microbiome from Infected and Non-Infected Salmonella pullorum Hens
Source: Microorganisms. 2025 Mar 11;13(3):640. doi: 10.3390/microorganisms13030640 (PMC11946299; doi:10.3390/microorganisms13030640)
Supplement: Supplementary file 1 [file microorganisms-13-00640-s001.zip › microorganisms-3514671-supplementary.pdf]

Table S1 Composition of experimental chicken diet.

Diet composition	Content (%)
Corn	65
Soybean meal	29
Fish meal	1
Premix[1]	5
**Diet chemical composition**	**Content**
Crude protein (%)	18.78
Metabolizable energy (Mcal/kg)	2.75
Crude fiber (%)	2.91
Ether extract (%)	3.90
Calcium (%)	0.91
Total phosphorus (%)	0.65
Sodium chloride	0.42
Lysine (%)	0.98
Methionine (%)	0.32
Cystine	0.33
Threonine	0.73
Tryptophan	0.22

[1]Premix: VA (IUkg): 165000; VD3 (IU/kg): 60000; VE (IU/kg): 600; VK3 (mg/kg): 60; VBI (mg/kg): 40; VB2 (mg/kg): 130, VB6 (mg/kg): 70; VB12 (mg/kg): 0.375; Niacin (mg/kg): 650; Pantothenic acid (mg/kg): 250; Folic acid (mg/kg): 22.5; Biotin (mg/kg): 1.75; Iron (mg/kg): 1300; Copper (mg/kg): 220; Manganese (mg/kg): 2450; Zinc (mg/kg):2200; Iodine (mg/kg):20; Selenium (mg/kg): 6.

Table S2 Alpha diversity indices among PN, PP and CT on14 days post-oral administration.

Indexes	PN_2w	PP_2w	CT_2w
Simpson	0.27±0.05 ^{a}	0.16±0.02 ^{b}	0.16±0.02 ^{b}
Chao	171.25±42.19 ^{a}	110.13±29.65 ^{ab}	79.50±14.31 ^{b}
ACE	171.25±42.19 ^{a}	110.13±29.65 ^{ab}	79.50±14.31 ^{b}
Sobs	171.25±42.19 ^{a}	110.13±29.65 ^{ab}	79.50±14.31 ^{b}

Note: ab, the significance level of the mean difference was 0.05. AB, the significance level of the mean difference was 0.01. PN: *S.* Pullorum-negative transplantation team, PP: *S.* Pullorum-positive transplantation team, CT: control team.

Table S3 Comparisons of taxonomic data at genus level among PN, PP and CT using One-way ANOVA.

Genera	PN	PP	CT
Lactobacillus	30274.6±1929.3 ^{a}	21448.5±3249.7 ^{b}	24462.8±1674.7 ^{ab}
Bacillus	482.6±149.6 ^{b}	2421.5±864.3 ^{a}	838.0±287.2 ^{ab}
Veillonella	79.1±62.9 ^{ab}	6.1±5.1 ^{b}	328.6±140.5 ^{a}
Phascolarctobacterium	18.4±9.9 ^{a}	0.3±0.3 ^{b}	1.1±1.1 ^{b}
norank_f__norank_o__Gastranaerophilales	51.1±21.4 ^{Aa}	2.8±1.8 ^{b}	0.5±0.5 ^{B}
norank_f__norank_o__Rhodospirillales	24.3±12.6 ^{a}	0.0±0.0 ^{b}	0.5±0.5 ^{b}

Note: ab, the significance level of the mean difference was 0.05. AB, the significance level of the mean difference was 0.01. PN: *S.* Pullorum-negative transplantation team, PP: *S.* Pullorum-positive transplantation team, CT: control team.

Table S4 The prediction functions with significant differences between different groups.

Prediction functions	Pathway_ID	Present study			Previous study [1]	
		PN	PP	CT	PP_2023	PN_2023
Glycolysis / Gluconeogenesis	ko00010	—	—	—	964287.3±34876.5 ^{B}	1351271.0±47231.2 ^{A}
Citrate cycle (TCA cycle)	ko00020	324813.9±22437.6 ^{a}	269500.9±19459.3 ^{ab}	245857.9±16959.0 ^{b}	530051.6±22180.7 ^{B}	769244.5±27737.1 ^{A}
Pentose phosphate pathway	ko00030	575784.3±24953.5 ^{a}	547850.3±35861.5 ^{ab}	479095.2±32655.2 ^{b}	625690.7±23147.5 ^{B}	843079.8±28299.1 ^{A}
Pentose and glucuronate interconversions	ko00040	169369.6±10650.8 ^{a}	158241.5±12383.6 ^{ab}	128604.9±13820.5 ^{b}	230971.7±7571.7 ^{B}	315537.2±12200.7 ^{A}
Fructose and mannose metabolism	ko00051	702415.5±28831.1 ^{a}	589679.0±31712.4 ^{b}	593069.4±46102.2 ^{b}	607976.0±20680.3 ^{B}	859941.0±31375.7 ^{A}
Galactose metabolism	ko00052	—	—	—	610800.1±21614.4 ^{B}	856093.3±33254.6 ^{A}
Fatty acid biosynthesis	ko00061	476625.9±39210.7 ^{A}	263737.6±25358.5 ^{B}	305155.3±20826.4 ^{B}	461452.1±17852.9 ^{B}	636830.1±21006.1 ^{A}
Fatty acid degradation	ko00071	77870.0±6797.4 ^{B}	117029.4±10557.5 ^{A}	76350.2±10373.5 ^{B}	143357.1±5701.8 ^{B}	193084.5±6782.7 ^{A}
Ubiquinone and other terpenoid-quinone biosynthesis	ko00130	—	—	—	130578.2±6575.2 ^{B}	212790.9±9211.9 ^{A}
Oxidative phosphorylation	ko00190	583901.0±31722.5 ^{a}	568401.4±39885.1 ^{ab}	467788.7±39247.8 ^{b}	909138.8±33920.2 ^{B}	1293818.1±45139.2 ^{A}
Photosynthesis	ko00195	296661.4±15273.3 ^{a}	256643.1±19081.1 ^{ab}	241511.6±13343.9 ^{b}	295756.7±11838.2 ^{B}	426178.6±14983.4 ^{A}
Arginine biosynthesis	ko00220	—	—	—	429001.5±16235.6 ^{B}	601660.7±20332.3 ^{A}
Purine metabolism	ko00230	1473841.5±77010.6 ^{A}	1163452.8±73743.4 ^{B}	1129238.4±70792.1 ^{B}	1368302.6±50423.7 ^{B}	1940875.5±67382.8 ^{A}
Pyrimidine metabolism	ko00240	1002217.6±47247.5 ^{a}	891057.2±62305.7 ^{ab}	804104.0±51021.5 ^{b}	1065535.7±39137.1 ^{B}	1492064.4±51565.9 ^{A}
Alanine, aspartate and glutamate metabolism	ko00250	609543.6±33263.6 ^{a}	557690.8±41993.9 ^{ab}	479939.1±31310.2 ^{b}	816370.3±29993.6 ^{B}	1163238.7±40434.7 ^{A}
Glycine, serine and threonine metabolism	ko00260	563736.8±31239.3 ^{a}	503175.1±35503.1 ^{ab}	448602.5±29522.2 ^{b}	782044.1±27975.8 ^{B}	1118096.4±39305.3 ^{A}
Monobactam biosynthesis	ko00261	151559.6±11318.2 ^{A}	125669.4±11612.9 ^{AB}	102167.6±5494.8 ^{B}	216244.5±7913.0 ^{B}	302430.7±10416.8 ^{A}
Cysteine and methionine metabolism	ko00270	655111.4±42708.9 ^{Aa}	526605.9±46114.0 ^{b}	478508.4±26718.9 ^{B}	833889.5±31055.5 ^{B}	1180714.4±40683.5 ^{A}
Valine, leucine and isoleucine degradation	ko00280	156370.3±9782.5 ^{Aa}	125788.8±9191.8 ^{b}	113621.7±7274.3 ^{B}	193067.1±7990.3 ^{B}	271177.1±9904.1 ^{A}
Valine, leucine and isoleucine biosynthesis	ko00290	—	—	—	372104.2±13904.6 ^{B}	531099.7±17885.0 ^{A}
Lysine biosynthesis	ko00300	476427.3±28467.6 ^{A}	413567.4±36871.6 ^{AB}	350223.0±17423.0 ^{B}	500237.3±18701.6 ^{B}	701399.0±23993.6 ^{A}
Arginine and proline metabolism	ko00330	240342.4±15667.2 ^{A}	198529.7±17548.5 ^{AB}	163010.3±13162.1 ^{B}	309308.3±11950.7 ^{B}	435517.7±14622.1 ^{A}
Prodigiosin biosynthesis	ko00333	156820.2±12767.9 ^{A}	85612.7±8167.8 ^{AB}	100198.8±6484.4 ^{B}	143498.7±5196.2 ^{B}	201026.7±6704.4 ^{A}
Histidine metabolism	ko00340	—	—	—	385767.1±14875.2 ^{B}	537341.1±18090.4 ^{A}
Tyrosine metabolism	ko00350	—	—	—	133152.0±4760.1 ^{B}	195315.2±7060.9 ^{A}
Phenylalanine metabolism	ko00360	—	—	—	163009.9±5903.4 ^{B}	246697.0±9523.9 ^{A}

Phenylalanine, tyrosine and tryptophan biosynthesis	ko00400	—	—	—	642452.1±23444.7 ^{B}	923993.5±32167.2 ^{A}
Selenocompound metabolism	ko00450	236719.0±12562.2 ^{Aa}	195870.5±11014.4 ^{B}	182581.1±12868.0 ^{b}	264230.7±9864.8 ^{B}	364362.1±12290.0 ^{A}
Cyanoamino acid metabolism	ko00460	121133.6±7280.2 ^{a}	110013.5±8759.1 ^{ab}	92418.1±6620.1 ^{b}	179143.5±6502.8 ^{B}	255316.1±10778.4 ^{A}
Starch and sucrose metabolism	ko00500	—	—	—	788138.7±29963.2 ^{B}	1062254.5±39406.7 ^{A}
Other glycan degradation	ko00511	—	—	—	234478.1±11433.2 ^{B}	349991.4±18506.2 ^{A}
Amino sugar and nucleotide sugar metabolism	ko00520	1126890.2±46863.8 ^{a}	950176.2±52839.9 ^{b}	922483.4±59425.2 ^{b}	1032851.1±37328.7 ^{B}	1458667.1±50936.8 ^{A}
Streptomycin biosynthesis	ko00521	—	—	—	259202.3±9249.8 ^{B}	358747.8±12591.2 ^{A}
Lipopolysaccharide biosynthesis	ko00540	—	—	—	308994.3±17646.1 ^{B}	474258.8±20251.7 ^{A}
Peptidoglycan biosynthesis	ko00550	721508.9±34178.7 ^{A}	629403.5±40584.3 ^{AB}	572485.2±33128.0 ^{B}	713326.8±26858.2 ^{B}	998303.2±33664.3 ^{A}
Glycerolipid metabolism	ko00561	416435.1±19267.9 ^{Aa}	338845.6±26734.8 ^{b}	322464.3±20441.3 ^{B}	251687.4±10205.8 ^{B}	341138.9±11942.7 ^{A}
Glycerophospholipid metabolism	ko00564	—	—	—	362360.9±13829.3 ^{B}	511637.3±17146.4 ^{A}
Sphingolipid metabolism	ko00600	—	—	—	137959.7±5774.1 ^{B}	200954.9±9792.1 ^{A}
Pyruvate metabolism	ko00620	891584.4±42900.3 ^{Aa}	752863.8±49201.9 ^{b}	693968.0±41083.6 ^{B}	845905.0±33737.2 ^{B}	1197626.3±40859.4 ^{A}
Glyoxylate and dicarboxylate metabolism	ko00630	—	—	—	609750.9±22156.3 ^{B}	906825.8±32725.1 ^{A}
Propanoate metabolism	ko00640	427773.2±23301.8 ^{A}	363984.3±24708.3 ^{AB}	325983.6±23297.4 ^{B}	466670.2±18820.1 ^{B}	658196.8±22755.3 ^{A}
Butanoate metabolism	ko00650	378808.4±19611.8 ^{a}	351380.2±24783.3 ^{ab}	297605.6±22743.8 ^{b}	482331.5±19155.1 ^{B}	698763.1±25420.9 ^{A}
C5-Branched dibasic acid metabolism	ko00660	—	—	—	214488.0±8159.6 ^{B}	308464.0±10620.8 ^{A}
One carbon pool by folate	ko00670	342540.6±13983.2 ^{Aa}	285013.9±17523.6 ^{B}	303831.2±20310.2 ^{b}	449817.9±16150.8 ^{B}	643472.6±23038.5 ^{A}
Methane metabolism	ko00680	563559.0±21414.3 ^{a}	526875.2±31601.5 ^{ab}	472941.5±33182.9 ^{b}	623677.5±23327.1 ^{B}	912953.7±31549.3 ^{A}
Carbon fixation in photosynthetic organisms	ko00710	345631.8±16622.4 ^{a}	333787.0±20716.6 ^{ab}	288624.0±19710.9 ^{b}	450965.8±16487.2 ^{B}	625737.3±21424.5 ^{A}
Carbon fixation pathways in prokaryotes	ko00720	539983.8±34377.1 ^{A}	468385.1±39348.8 ^{AB}	404389.9±22342.4 ^{B}	828915.1±33425.1 ^{B}	1202535.9±42358.1 ^{A}
Thiamine metabolism	ko00730	304793.1±16093.2 ^{a}	301535.5±25921.5 ^{ab}	245595.6±13612.7 ^{b}	411077.4±15421.8 ^{B}	593070.6±20747.7 ^{A}
Riboflavin metabolism	ko00740	—	—	—	166489.6±6159.6 ^{B}	239004.3±8586.7 ^{A}
Vitamin B6 metabolism	ko00750	—	—	—	156314.4±5500.8 ^{B}	223816.8±8376.8 ^{A}
Nicotinate and nicotinamide metabolism	ko00760	293105.3±15510.7 ^{a}	273227.9±18205.9 ^{ab}	232899.4±18011.2 ^{b}	387936.4±13941.9 ^{B}	553233.2±19124.7 ^{A}
Pantothenate and CoA biosynthesis	ko00770	324562.6±20681.4 ^{a}	279610.1±22911.8 ^{ab}	247904.6±18696.4 ^{b}	484906.7±17485.6 ^{B}	693859.3±23890.2 ^{A}
Biotin metabolism	ko00780	276318.3±22390.7 ^{A}	162581.1±16137.8 ^{B}	176912.6±14554.7 ^{B}	345544.2±12792.3 ^{B}	501976.0±17980.5 ^{A}
Folate biosynthesis	ko00790	—	—	—	392776.7±14436.8 ^{B}	574752.8±21116.5 ^{A}
Porphyrin and chlorophyll metabolism	ko00860	—	—	—	586379.2±25637.6 ^{B}	862326.5±31192.2 ^{A}
Terpenoid backbone biosynthesis	ko00900	394148.2±16791.3 ^{a}	363794.6±27522.3 ^{ab}	323690.6±19203.1 ^{b}	413782.9±16006.2 ^{B}	578386.3±19805.2 ^{A}
Nitrogen metabolism	ko00910	—	—	—	281053.1±10699.7 ^{B}	409667.4±14315.1 $^{A\ A}$

Sulfur metabolism	ko00920	—	—	—	244817.5±11231.2 ^{B}	376591.2±14954.8 ^{A}
Aminoacyl-tRNA biosynthesis	ko00970	944301.6±44165.1 ^{a}	820544.0±56361.2 ^{ab}	757517.1±42184.3 ^{b}	916662.6±35508.4 ^{B}	1296530.7±44085.7 ^{A}
Drug metabolism - other enzymes	ko00983	278209.3±12438.5 ^{a}	254101.7±16164.2 ^{ab}	223742.3±15425.0 ^{b}	313736.6±11306.6 ^{B}	433688.9±15271.6 ^{A}
Metabolic pathways	ko01100	11724331.1±619154.9 ^{a}	10551870.3±690142.7 ^{ab}	9312611.8±659266.1 ^{b}	15429732.0±564018.2 ^{B}	21964254.0±761561.2 ^{A}
Biosynthesis of secondary metabolites	ko01110	5395661.1±316298.8 ^{a}	4745541.3±353335.5 ^{ab}	4209255.5±276427.3 ^{b}	7496146.0±274495.8 ^{B}	10663255.2±367116.9 ^{A}
Microbial metabolism in diverse environments	ko01120	2972806.5±145536.7 ^{a}	2727284.0±171288.0 ^{ab}	2374097.3±180920.2 ^{b}	3593076.3±135522.4 ^{B}	5131378.6±176856.1 ^{A}
Carbon metabolism	ko01200	1823742.9±94874.7 ^{a}	1620779.4±106267.7 ^{ab}	1448067.4±92319.2 ^{b}	2310537.2±87787.9 ^{B}	3273960.4±111429.8 ^{A}
2-Oxocarboxylic acid metabolism	ko01210	—	—	—	724091.2±27126.2 ^{B}	1028770.9±34725.3 ^{A}
Fatty acid metabolism	ko01212	462130.2±36723.7 ^{A}	273649.0±26506.8 ^{B}	296839.3±20515.2 ^{B}	485839.4±19117.1 ^{B}	667253.9±22025.3 ^{A}
Biosynthesis of amino acids	ko01230	—	—	—	3536559.0±128984.5 ^{B}	5000102.7±169699.6 ^{A}
beta-Lactam resistance	ko01501	418155.9±21987.8 ^{Aa}	345729.1±23856.4 ^{b}	313289.6±19183.9 ^{B}	380283.6±13584.2 ^{B}	546671.6±20172.7 ^{A}
Vancomycin resistance	ko01502	228906.8±11935.9 ^{Aa}	192758.9±13033.1 ^{b}	173639.5±10163.8 ^{B}	235183.5±9045.4 ^{B}	330589.1±11315.1 ^{A}
Cationic antimicrobial peptide (CAMP) resistance	ko01503	—	—	—	237754.3±8789.3 ^{B}	360061.9±14453.6 ^{A}
Antifolate resistance	ko01523	147991.6±8626.6 ^{Aa}	120093.3±6934.9 ^{b}	114197.7±7346.0 ^{B}	171010.1±5989.1 ^{B}	245049.8±9053.9 ^{A}
ABC transporters	ko02010	—	—	—	1751389.5±84145.3 ^{B}	2332438.4±79102.7 ^{A}
Two-component system	ko02020	1121138.5±45431.9 ^{ab}	1196210.0±94382.9 ^{a}	938162.5±99856.3 ^{b}	1207722.5±65545.6 ^{B}	1690391.6±61583.1 ^{A}
Quorum sensing	ko02024	—	—	—	1041957.0±45380.1 ^{B}	1447154.3±48784.5 ^{A}
Biofilm formation - Escherichia coli	ko02026	101425.8±15278.6 ^{ab}	151519.5±17894.1 ^{a}	100223.0±18448.8 ^{b}	235987.6±9278.6 ^{B}	327834.6±11036.5 ^{A}
Bacterial chemotaxis	ko02030	—	—	—	360009.6±36732.9 ^{b}	458213.5±24422.8 ^{a}
Flagellar assembly	ko02040	113880.1±31494.5 ^{ab}	180301.2±36003.1 ^{a}	74779.7±32292.6 ^{b}	370458.1±38720.6 ^{B}	502875.6±27687.1 ^{A}
Phosphotransferase system (PTS)	ko02060	—	—	—	308138.3±18967.5 ^{B}	395748.6±20927.3 ^{A}
Ribosome	ko03010	2040624.8±93731.1 ^{a}	1779058.4±119879.9 ^{ab}	1645192.2±92172.8 ^{b}	2022476.2±76620.1 ^{B}	2864588.1±98212.2 ^{A}
RNA degradation	ko03018	433958.0±22232.7 ^{a}	385381.4±25588.8 ^{ab}	345403.7±20962.5 ^{b}	529695.3±19684.1 ^{B}	747439.2±25285.2 ^{A}
DNA replication	ko03030	548372.0±21859.4 ^{a}	499670.9±34370.5 ^{ab}	455809.2±25519.8 ^{b}	552799.8±20778.0 ^{B}	785926.6±27105.5 ^{A}
Protein export	ko03060	445936.8±19194.7 ^{a}	411805.7±25194.9 ^{ab}	372713.8±23671.5 ^{b}	518401.5±19655.7 ^{B}	740904.4±25500.9 ^{A}
Bacterial secretion system	ko03070	—	—	—	508258.9±20962.6 ^{B}	760339.0±26806.2 ^{A}
Base excision repair	ko03410	342540.6±13983.2 ^{a}	303831.2±20310.2 ^{ab}	285013.9±17523.6 ^{b}	306818.8±11828.0 ^{B}	428899.6±14650.0 ^{A}
Nucleotide excision repair	ko03420	314991.8±13649.0 ^{a}	290899.8±21561.2 ^{ab}	259211.3±14842.1 ^{b}	319154.8±12041.6 ^{B}	447156.6±15187.8 ^{A}
Mismatch repair	ko03430	661268.1±26976.2 ^{a}	600929.3±39659.3 ^{ab}	543650.6±31527.7 ^{b}	658718.3±24935.1 ^{B}	934466.5±32020.4 ^{A}
Homologous recombination	ko03440	745761.1±31693.2 ^{a}	685324.9±46602.1 ^{ab}	614760.5±36724.8 ^{b}	780940.4±29163.6 ^{B}	1106679.7±38330.5 ^{A}
HIF-1 signaling pathway	ko04066	287643.0±13152.2 ^{a}	246858.2±18427.4 ^{ab}	232786.8±13703.4 ^{b}	227132.0±8376.3 ^{B}	314587.0±10801.2 ^{A}

Cell cycle - Caulobacter	ko04112	387590.6±17407.6 ^{A}	343761.0±21748.6 ^{AB}	310170.9±16920.9 ^{B}	425543.1±15796.4 ^{B}	606052.3±20635.1 ^{A}
Sulfur relay system	ko04122	—	—	—	202405.1±8613.4 ^{B}	290609.8±9965.4 ^{A}
Lysosome	ko04142	—	—	—	145715.1±6888.0 ^{B}	219818.2±11600.3 ^{A}
Peroxisome	ko04146	—	—	—	147510.0±6070.1 ^{B}	209522.4±7798.1 ^{A}
Longevity regulating pathway - worm	ko04212	126283.0±4878.1 ^{a}	119468.0±6388.9 ^{ab}	105407.3±7382.4 ^{b}	153294.2±5930.4 ^{B}	215928.2±7297.2 ^{A}
Necroptosis	ko04217	—	—	—	150897.1±5528.5 ^{B}	214693.9±7543.3 ^{A}
Plant-pathogen interaction	ko04626	91696.8±6397.2 ^{Aa}	87666.2±8615.7 ^{b}	66013.6±4463.9 ^{B}	156452.7±7267.8 ^{B}	216755.6±7241.4 ^{A}
Glucagon signaling pathway	ko04922	286914.3±12061.2 ^{a}	250096.1±18803.4 ^{ab}	238229.1±13815.6 ^{b}	208547.0±7848.8 ^{B}	282601.3±9742.7 ^{A}
Biofilm formation - Vibrio cholerae	ko05111	—	—	—	239046.0±11301.4 ^{B}	351676.4±13610.1 ^{A}
Salmonella infection	ko05132	122556.6±7260.4	103150.7±8703.7 ^{ab}	87851.8±5270.0 ^{b}	173431.2±8124.0 ^{B}	245288.1±8195.4 ^{A}
Central carbon metabolism in cancer	ko05230	—	—	—	169504.3±6559.8 ^{B}	225697.1±7950.5 ^{A}
Fluid shear stress and atherosclerosis	ko05418	109219.6±8309.2 ^{A}	76447.6±7881.1 ^{B}	73403.9±5184.1 ^{B}	141830.4±5372.4 ^{B}	199313.3±6715.3 ^{A}
Taurine and hypotaurine metabolism	ko00430	108820.4±5150.3 ^{A}	84720.3±4197.3 ^{B}	85477.4±5170.9 ^{B}	—	—
D-Glutamine and D-glutamate metabolism	ko00471	120705.2±4724.1 ^{a}	111441.2±7278.0 ^{ab}	100716.8±6677.4 ^{b}	—	—
D-Alanine metabolism	ko00473	144428.5±5828.1 ^{a}	123875.1±11644.2 ^{ab}	118165.3±6690.8 ^{b}	—	—
Glutathione metabolism	ko00480	245253.0±11097.6 ^{a}	195077.1±17499.3 ^{ab}	202603.9±17677.9 ^{b}	—	—
RNA polymerase	ko03020	181609.4±7894.9 ^{a}	155699.7±12227.8 ^{ab}	148008.3±8347.1 ^{b}	—	—
Legionellosis	ko05134	86827.9±3986.5	81081.1±4878.7 ^{AB}	67638.8±4876.0 ^{B}	—	—
Staphylococcus aureus infection	ko05150	156519.6±9628.5 ^{a}	114315.4±18330.0 ^{ab}	127240.0±5585.6 ^{b}	—	—
Tuberculosis	ko05152	114534.7±5190.8 ^{a}	100758.5±6621.0 ^{ab}	92634.9±5376.1 ^{b}	—	—

[1] study of Niu et al.. PP_2023: S. Pullorum-negative hens team in Niu et al., PN_2023: S. Pullorum- positive hens team in Niu et al., PN: S. Pullorum-negative transplantation team, PP: S. Pullorum-positive transplantation team, CT: control team.
